# A fixed nitrous oxide/oxygen mixture as an analgesic for trauma patients in emergency department: study protocol for a randomized, controlled trial

**DOI:** 10.1186/s13063-018-2899-6

**Published:** 2018-09-29

**Authors:** Lu-Lu Gao, Li-Shan Yang, Jun-Jun Zhang, Yi-Ling Wang, Ke Feng, Lei Ma, Yuan-Yuan Yu, Qiang Li, Qing-Huan Wang, Jin-Tao Bao, Ya-Liang Dai, Qiang Liu, Yu-Xiang Li, Qiang-Jian Yu

**Affiliations:** 1 School of Nursing, Ningxia Medical University, 1160 Shengli Street, Yinchuan, 750004 China; 2 Emergency Department, General Hospital of Ningxia Medical University, Yinchuan, 750004 China; 3 School of Basic Medical Sciences, Ningxia Medical University, 1160 Shengli Street, Yinchuan, 750004 China; 4 Institute of Nursing Research, Ningxia Medical University, 1160 Shengli Street, Yinchuan, 750004 China; 5 Department of Pharmacology, Pharmaceutical Institute of Ningxia Medical University, 1160 Shengli Street, Yinchuan, 750004 China

**Keywords:** Trauma, Analgesia, Acute pain, Nitrous oxide

## Abstract

**Background:**

Acute pain is always the most common complaint in Emergency Department admissions and options for analgesia are limited. Nitrous oxide/oxygen possess many properties showing it may be an ideal analgesic method for the Emergency Department; it is quick-acting, well-tolerated, and does not mask signs and symptoms. The aim of this study is to evaluate the safety and analgesic effect of the fixed nitrous oxide/oxygen mixture for trauma patients in a busy emergency environment.

**Methods:**

The randomized, double-blind, prospective, placebo-controlled study will be carried out in the Emergency Department of General Hospital of Ningxia Medical University. The target research objects are trauma patients who present to the Emergency Department and report moderate to severe intensities of acute pain. A total of 90 patients will be recruited and randomly assigned into the treatment and control group. The treatment group will receive conventional pain treatment plus nitrous oxide/oxygen mixture and the control group will receive conventional pain treatment plus oxygen. Neither patients, nor investigators, nor data collectors will know the nature of the gas mixture in each cylinder and the randomization list. Outcomes will be monitored at baseline(T0), 5 min (T1), and 15 min (T2) after the beginning of intervention and at 5 min post intervention (T3) for each group. The primary outcome is the level of pain relief after the initial administering of the intervention at T1, T2, and T3. Secondary outcomes include adverse events, physiological parameters, total time of the gas administration, satisfaction from both patients and healthcare professionals, and the acceptance of patients.

**Discussion:**

Our previous studies suggested that a fixed nitrous oxide/oxygen mixture was an efficacious analgesic for the management of burning dressing pain and breakthrough cancer pain. The results of this study will provide a more in-depth understanding of the effect of this gas. If this treatment proves successful, it could help to generate preliminary guidelines and be implemented widely in trauma patients with pain in Emergency Departments.

**Trial registration:**

Chinese Clinical Trial Register, ChiCTR-INR-16007807. Registered on 21 January 2016.

**Electronic supplementary material:**

The online version of this article (10.1186/s13063-018-2899-6) contains supplementary material, which is available to authorized users.

## Background

Despite there are extensive research on the etiology of pain, barriers to pain management, advanced analgesia approach, and evidence-based guidelines for the pain management such as the World Health Organization three-step analgesic ladder, acute pain remains a common complaint observed in the Emergency Department (ED) with a high prevalence of up to 90% [1–3]. Over the past decades, emergency health workers have documented that the prevalence of pain undertreatment is high. Numerous studies demonstrated that 60–80% of patients reported pain on admission to the ED [2, 4, 5]. It continues to be a challenge for emergency healthcare givers to treat oligoanalgesia or undertreatment of pain [6]. A prospective cross-sectional study assessed 525 ED patients’ experiences and satisfaction with pain management and reported that only half of patients received analgesics even though 63% suffered with severe pain. The average time to analgesic administration was almost 2 h and the percentage of patients who received analgesics within 1 h after arrival was only 29% [7]. In a retrospective medical record study, Wilson and Pendelton [8] found that no more than half of patients admitted to the ED for acute pain were treated with analgesics during hospitalization. Of these, nearly 70% of patient waited > 1 h and > 40% waited > 2 h for analgesics. In another prospective study conducted in the ED of a teaching hospital in Central Africa, nearly 50% of the patients did not receive prescribed analgesia before definitive surgical treatment, including most patients with severe pain [9]. These observed results are consistent with previous studies [10, 11]. Taking into account previous reports, the results of these studies show that the practice of oligoanalgesia in the ED is a global phenomenon, partially because emergency health workers fail to fully assess or perceive the patient’s pain intensity [9]. All in all, the percentage of patients who receive any type of analgesia and pain relief is still deficient.

Efforts to treat acute pain are priorities for ED healthcare to consider. Patients expect analgesia rapidly in the ED, which is not met in many ED [12]. Several reasons have been related to poorly managed pain. First, the failure assessment or lack of skills to assess patients’ pain is a main predicting factor of pain treatment deficiency. The physicians’ underestimation of pain can be circumvented by using the pain assessment scales [13–15]. Second, in our institution, no analgesic guideline exists to help the physician prescribe analgesics for patients in need and no effort was made to influence physicians’ orders for analgesia [4]. Physicians are also worried about concealing, delaying, or obscuring diagnosis [11, 16], although there is poor evidence of this. There are still many myths and misconceptions about opioids, addiction, and tolerance [17, 18]. We likewise have a limited ability to monitor the cardiopulmonary function of opiates in the ED. Third, a crowded ED setting and insufficient ED staff affect acute pain assessment and reassessment [19]. It is probable that busy healthcare professionals pay less attention to complaints of painful conditions. In fact, pain management is not a priority in assessing and resuscitating these patients traditionally. Other factors including gender, race, age, education, language, and cultural and socioeconomic backgrounds also play important roles in the disparity of treatment [20–23].

However, patients with unrelieved pain lead to a stress response consisting of increased heart rate and blood pressure, impaired immune function, systemic vascular resistance, and altered release of neuroendocrine, pituitary, and other hormones [24]. These responses could limit patients’ recovery from injury, resulting in longer hospitalization, higher readmission rates, and more frequent outpatient visits. These factors may lead to lower rates of ED patient satisfaction and an increase in medical cost [25]. Hence, acute pain management should be a priority for us to consider and we have several reasons to search for safe and universal analgesics.

Nitrous oxide/oxygen is a self-administered inhaled gas reserved in a pre-prepared cylinder. Its analgesic effect has been known for nearly two centuries [26]. It has few side effects, all of which disappear after termination of exposure to the gas. There is no need for an endovenous procedure. The patients’ self-sustained facemasks lower the risk of overdose as the patients’ level of consciousness control their ability to maintain the gas flow [27]. Therefore, it is safe and non-invasive [28]. Owing to its low solubility in blood and not bound to proteins, it possesses the properties of rapid onset (no more than 2 min) and is quickly reversible after discontinuation (< 1 min). As a result, nitrous oxide does not obscure the signs and symptoms that may be necessary for subsequent damage or disease definitive diagnosis. It is reported that the mixture will play the same role as of a 15-mg dose of morphine given intramuscularly [29]. These properties also suggest that nitrous oxide/oxygen may be an ideal analgesic for ED use. Taking these advantages into account, we hypothesize that a fixed concentration of nitrous oxide/oxygen mixture (Patent no. ZL 2013 1 0053336.X) can provide superior analgesic effects for trauma patients with acute pain in the ED.

## Methods/design

### Aim

The primary aim of the current study is to determine the clinical analgesic effect of the fixed nitrous oxide/oxygen mixture on trauma patients with acute pain when compared with the control group. It is measured by pain relief level. Secondary objectives of this trial include:

1. Evaluating the clinical use safety of the fixed gas by physiological parameters and adverse effects.
2. Assessing the satisfaction of both patients and healthcare workers with pain treatment.
3. Investigating patients’ acceptance for the new analgesia method.

### Study design

**Fig. 1:**
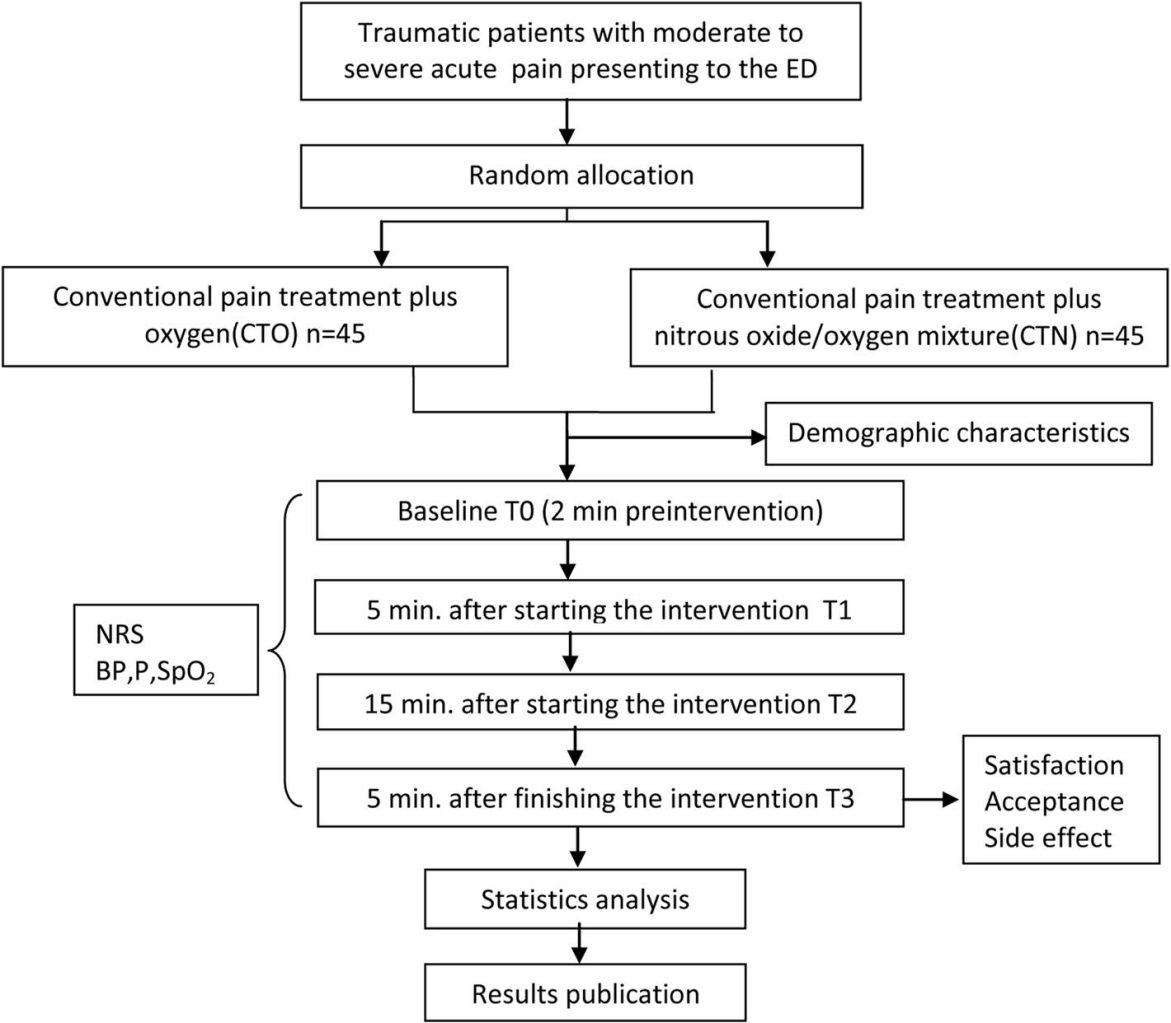
Study design. NRS numerical pain rating scale, BP blood pressure, HR heart rate, SpO_2_ oxygen saturation, CTO conventional pain treatment plus oxygen, CTN conventional pain treatment plus nitrous oxide/oxygen mixture

**Fig. 2:**
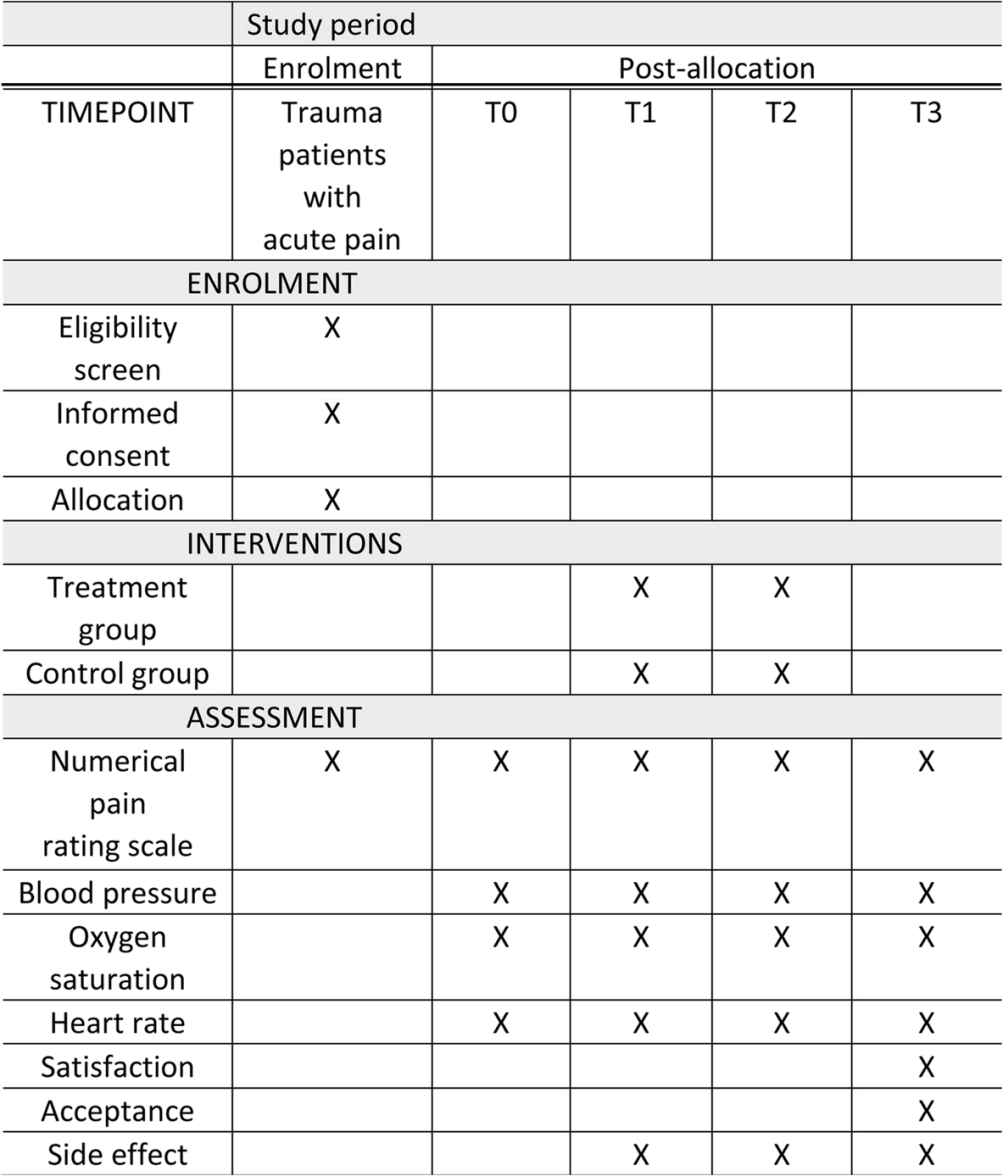
SPIRIT *figure*: schedule of enrolment, interventions, and assessments

This is a single-center, prospective, randomized, double-blind, placebo-controlled, two-arm, superiority study. It was designed to test the analgesic effect and safety of inhaling premixed nitrous oxide/oxygen use in adult trauma patients with moderate to severe acute pain. This protocol was drafted using the SPIRIT guidelines [30] (see Additional file 1) and following the checklist of the Consolidated Standards of Reporting Trials (CONSORT) statement (see Additional file 2). The clinical trial was reviewed and approved by the Ningxia Medical University Ethics Committee. This study has been registered at Chinese Clinical Trial Registry (registration no. ChiCTR-INR-16007807). The whole study design is provided in Fig. 1. Additionally, a SPIRIT figure for the schedule of enrolment, interventions, and assessments is presented in Fig. 2.

### Study setting

This study will be carried out in the ED of General Hospital of Ningxia Medical University. This institution is an urban, public, 4500-bed, tertiary-care teaching hospital located in Northwest China, with an ED census of 179,117 annually.

### Study participants

All trauma patients who present to the ED will be invited to participate. The specific inclusion criteria are: patients who speak Chinese; aged ≥ 18 years; report moderate to severe intensity of acute pain upon admission (defined as a pain score ≥ 4 according to the Numerical Rating Scale [NRS]); are able to take deep breaths to use the self-managed device; are willing to participate in the study and sign informed consent. Patients are ineligible if they have considered contraindications to using the premixed gas (intracranial hypertension, recent ophthalmic surgery, epilepsy, unconsciousness, pneumothorax / hemothorax, facial traumas, intestinal obstruction, pulmonary embolism, emphysema, sinusitis, and retinal detachment [17]). Other exclusion criteria are: recent receipt of analgesics within 6 h; life-threatening situations or instability of clinical vital signs; pregnancy; individuals in police custody; or individuals included in another study.

All participating clinicians will be associate chief physicians, will have worked in the ED for > 5 years, and will be willing to participate in and receive training from the originator who conceived and designed the protocol. In this trial, two fixed clinicians who meet all the above criteria will be included.

### Interventions

All patients entering the ED will receive regular assessments by the clinician. The patients who meet all pre-specified inclusion and exclusion criteria will be introduced to the study. Investigators will explain the purpose, benefits, and potential risks of the study in detail to the patients. If the patients agree to participate in this study, they will be asked to sign an informed consent form and then be recruited. Subsequently, the relevant treatment will be carried out immediately. During the implementation of the intervention, the patients are randomly allocated to inhale either a fixed concentration of premixed nitrous oxide/oxygen or oxygen through a specially designed facemask with a one-way valve. The treatment group will receive nitrous oxide/oxygen mixture in addition to conventional pain treatment and the control group will receive oxygen in addition to conventional pain treatment. In this study, the oxygen cylinder is designed exactly the same as the nitrous oxide cylinder and oxygen is safe and readily available in the selected ED. We chose oxygen plus conventional pain treatment as a comparator to ensure the full consistency between treatment group and control group and the double-blind design. Gas inhalation will be administered continuously up to a maximum inhalation time of 15 min. Practitioners will be permitted to use other pain management forms in accordance with patients’ pain intensity after finishing the intervention. The trained investigator will successively include participants until they reach the target sample size and the project manager will assign participants to interventions according to the randomization list.

Generally, both nitrous oxide/oxygen and oxygen are considered safe interventions with no severe side effects for the participants. If side effects occur (e.g. dizziness, nausea) during the implementation of nitrous oxide/oxygen, they are usually short-lived and mild [28]. If any undesirable events occur or the participant requests to cease the intervention they received, the investigator can discern whether the trial will be continued. In all cases, the reason for discontinuation will be documented by the data collector.

There are many strategies to improve the adherence of the intervention. Before the start of the intervention, investigators will detail the duration of the intervention, the method of using the device, possible adverse effects, and give psychological support to participants. During the treatment, the patients’ physiological parameters are monitored and patients are asked if they feel uncomfortable or not. If they are uncomfortable, this is address immediately to ensure the ongoing safety of patients is accomplished. Adherence of intervention protocols will be ascertained by accomplishment of the intervention. We planned to develop a Supervision Adherence Form (SAF) for this trial to enable collation of patients’ adherence to facilitate reporting.

### Randomization, allocation concealment, and blinding

All participants will be randomly allocated into the intervention or control group in a ratio of 1:1. The randomization list of each ED patient will be generated by a computer, which is calculated by a statistical expert from the Faculty of Medical Statistics and Epidemiology, Ningxia Medical University. A sealed envelope will be used to store the randomization schedule which will be restricted in a dedicated research office to maintain confidentiality. In order to make sure of “double-blind” conditions, no data collectors or investigators have access to the data distribution. Only the project manager who will be responsible for gas distribution can consult this information. Ningfeng Oxygen Company packages the two gas mixtures in strictly identical-looking gas cylinders, one containing nitrous oxide/oxygen and the other oxygen. Therefor, all the gas cylinders will be identified only with a label (A or B). Patients given the letter A will be treated using a canister with the appropriate letter containing premixed nitrous oxide/oxygen. Those with the letter B will be given oxygen. Before and during the treatment period, the patient, the investigator, and the data collector are also blind to what is in each canister and what the letters A and B stand for. The rules were that the cessation of blinding would take place by the project manager only, after the completion of all the trials.

### Measurement

The investigator will record participants’ characteristics, demographic data (age, sex, nationality, weight, and height), and physiological parameters (pulse rate, blood pressure, and oxygen saturation). Other information regarding pain score, diagnosis of traumatic injury, total length of gas inhalation, analgesic prescription, adverse effects, satisfaction of patients and healthcare professionals, as well as the acceptance from patients will be recorded by the researcher.

Self-assessment of pain severity will be measured with the NRS. The NRS is a tool with great reliability and validity that is frequently used for assessing pain level from zero (painfree) to ten (worst pain intolerable) [31]. The physical parameters will be monitored via an electronic manometer (OMRON, HEM-7120) and OXIMETER (PC-60B). The evaluated pain score along with non-invasive monitoring of blood pressure, heart rate, and digital monitoring of oxygen saturation at baseline (T0) before the intervention, 5 min (T1), and 15 min (T2) after the beginning of the intervention, and at 5 min (T3) after the intervention.

Any observed adverse effects of the fixed nitrous oxide/oxygen mixture and oxygen will be carefully evaluated and recorded by the investigator after the beginning of the gas administration. The prospectively occurred adverse effects include gastrointestinal adverse effects (vomiting, nausea), respiratory adverse effects (oxygen desaturation which defined as pulse oximetric saturation ≦ 94%, arterial hypotension, and bradycardia), and any other discomfort (numbness, dizziness, drowsiness, oversedation, and headache) [32]. If any adverse effects take place, patients will be given inhaled oxygen and in < 5 min the adverse effects will vanish. All severe adverse effects and management of them during the gas inhalation will also be noted.

Both patients’ and healthcare providers’ satisfaction with pain administration under the new analgesia will be investigated by a five-point satisfaction scale (5, very satisfied; 4, satisfied; 3, uncertain; 2, dissatisfied; 1, very dissatisfied) [33]. Moreover, the acceptance will be obtained by asking patients whether they would accept the same gas inhalation in case of another trauma with moderate to severe pain (yes / no). The satisfaction and the acceptance are recorded at T3.

The clinical demographic data, details of trauma type, and analgesic prescription will be obtained from medical records using a standardized case report form.

### Outcome measures

#### Primary outcome measure

The primary outcome measure will be the level of pain relief after the initiation of the intervention, which is assessed and recorded by the data collector according to the pain level provided by patients using a self-assessment scale (NRS).

#### Secondary outcome measures

Secondary outcome measures include physiological parameters, adverse events, overall satisfaction from both patients and healthcare professionals, acceptance from patients, and total time of the gas administration.

### Sample size determination

Based on our previous studies on cancer breakthrough pain and burn-dressing pain [32, 34], this study uses pain severity as the primary endpoint measure for sample size calculation. We used preliminary visits obtained in our ED for 20 patients and calculated the sample size at 5 min (T1) and 15 min (T2) after the beginning of intervention. It is observed that 65% of patients receiving premixed nitrous oxide/oxygen had pain relief at T1 compared with 8% of patients receiving oxygen (a decrease in pain intensity of at least 30% was recommended as the pain relief [35]). We found that 70% of patients in the experimental group experienced pain relief at T2 versus 10% in the control group. It aimed to determine a appropriate target sample size that would provide a 90% power (*β* = 0.10) for two-tailed testing with 5% type-1 error rate of the measure. We separately calculated a minimum requirement of 30 patients or 24 patients for total with the formula [36, 37]. In practice, we finally decided on 90 samples (45 per group) to improve the power of this study.

### Recruitment

In this trial, participants will be recruited at the ED of General Hospital of Ningxia Medical University. This medical center is the largest hospital in Ningxia Hui Autonomous Region. It is well-known in the northwestern region of China for its comprehensive disciplines, powerful technology, advanced medical equipment, outstanding specialties, and strong multidisciplinary advantages. Furthermoremore, according to the ED census data of 2016, there is a sufficient patient source in this hospital. Thus, in this selected clinical center, it is feasible to reach the target sample size.

### Participant retention and withdrawal

As soon as a participant is enrolled, the research team are responsible for achieving a low rate of loss to intervention. Patients have the right to withdraw from the study at any time and this will not affect their subsequent treatment. The Data Monitoring Committee (DMC) will discuss and analyze the reason of dropout with the data collectors, which will be documented on a standardized pro forma.

### Data collection, management, and confidentiality

All members involved in the research team will receive group training sessions before the study. Training will include study design (e.g. require double-blind), intervention process (e.g. inhaling gases via facemask), assessment of the outcome measures (e.g. standardized procedures to assess), and recorded data (e.g. training for the use of a unified data collection form in detail). Furthermore, the trained data collectors and investigators are fixed during the implementation of interventions and through the double-entry method for data quality control. The members of the DMC and the auditor will inspect this database regularly to promote data quality (see “Data monitoring” and “Auditing”). Using a Microsoft Access database (Microsoft Office 2010, Redmond, WA, USA) to accomplish all study data input.

Throughout the trial, participants are anonymized. All participants’ personal information (e.g. name and patient identification number) will be kept in a separate locked cabinet and will be entered into the password-protected Microsoft Access database. Only research team members can access this personal data with reasonable request and consent of the project manager. A separate code, generated by pre-randomization, will enable anonymized data to be identified and this unique identifier will be maintained by the project manager. Anonymized trial data will be stored securely in another Microsoft Access database. The trial data collectors, the investigator, and the members of the DMC can access the trial data for the final statistical analysis.

### Data monitoring

This trial will be overseen by a DMC, which is independent from the sponsor and competing interests. Members consisted of one clinician, two nurses working in the ED, one pain management specialist, and a senior academic statistician who acts as the committee’s chair. The DMC will meet regularly to monitor the trial progress and guarantee protocol adherence. They are also responsible for checking for the reason of the participant withdrawal from this trial, assessing safety issues, and if there are any reasons that the trial should discontinue or make amendments.

### Auditing

The trial funder will apply audits annually and inspect the source data to verify the accuracy, completion, validity, and safety of the data. The auditing will potentially include the utilization of funds, the essential documents, the trial progress, and the participants’ medical records.

### Data analysis

SPSS version 22.0 (Chicago, IL, USA) will be used to conduct the statistic analysis, initially according to the intention-to-treat principle. All patients randomized should be included in the analysis and missing data will be handled using multiple imputation. Descriptive statistics will be adopted to describe the population demographic data by medians (interquartile ranges [IQR]; min–max), means (standard deviations), and proportions (exact binomial 95% confidence intervals [CIs]), as appropriate. The comparison of means will be assessed by Student’s *t* test or non-parametric test and using the Mann–Whitney test to compare means of continuous variables if applicable. Chi-squared tests or Fisher’s exact test will be performed to compare the proportions of categorical variables. The analysis of physiological parameters and scores at baseline, T2, and T3 were performed by analysis of covariant (ANCOVA) adjusting for the respective baseline value on the two groups (“experimental” and “control”). We considered all *P* values < 0.05 to be statistically significant.

### Ethics and dissemination

The findings of this study will be shared with healthcare workers, patients, the general public, and relevant departments through open-access articles, public talks, conferences, and final reports. Any modifications of this protocol and changes to eligibility criteria or outcomes will be communicated with the DMC, clinicians, trial participants, and research ethics committee review board. There are no professional writers to be employed to complete the manuscript. Authorship eligibility guidelines which comply with the International Committee of Medical Journal Editors (ICMJE) guidelines [38] will be adhered to.

## Discussion

The commitment to manage a patient’s pain and relieve suffering is the foundation of a healthcare professional’s obligation. Pain is not just a clinical symptom but also an evidence of potential pathology. In acute trauma environments, there is a temptation to ignore pain and its unique management, but all treatments lay the attention on the underlying pathology [24]. The challenge is to help health workers realize that symptoms and potential pathophysiology should go hand in hand.

In trauma patients, we usually give priority to injury mechanisms and assess the patient’s signs of life, including the level of consciousness. Nevertheless, opioids may interfere with this assessment and necessitate continuous monitoring for some patients using these drugs [27, 39]. Opioids may also interact with other medicine used in the ED and should be administered cautiously [18]. It may be impractical to achieve completely painless in acute traumatic settings at present stage. However, by using widely used techniques, pain can be decreased to tolerable levels [24]. In fact, numerous studies compared the efficacy of nitrous oxide/oxygen with opioids, which showed that 50% nitrous oxide/oxygen decreased the overall opioids consumption in some foreign countries [9, 18, 40–42]. Furthermore, our previous studies have suggested that the appropriate concentration of diluted nitrous oxide managed by health workers for burn dressing and breakthrough pain are safe and effective [27, 32, 34].

To the best of our knowledge, this is the first randomized controlled trial in a busy emergency environment to evaluate the safety and analgesic effectiveness of the fixed nitrous oxide/oxygen mixture in patients with trauma in China. Pain is a natural accompaniment of acute injury to tissues, which is predictable in the case of acute trauma. If this treatment seems to be beneficial, this study could help to generate preliminary pain guidelines in patients with acute trauma and improve patients’ overall satisfaction with ED. We believe that the results of this study could be considered as the introduction of pain management and be disseminated to international journals and conferences.

### Trial status

Recruitment of patients began in November 2017.

## Additional files

Additional file 1: SPIRIT 2013 Checklist: Recommended items to address in a clinical trial protocol and related documents. (DOC 115 kb)

Additional file 2: CONSORT 2010 checklist of information to include when reporting a randomized trial. (DOC 195 kb)

## Abbreviations

ANCOVA: Analysis of covariant
CONSORT: Consolidated Standards of Reporting Trials
DMC: Data Monitoring Committee
ED: Emergency Department
ICMJE: International Committee of Medical Journal Editors
NRS: Numerical Rating Scale
SAF: Supervision Adherence Form
